# Dissolved Organic Matter Modulates Algal Oxidative Stress and Membrane System Responses to Binary Mixtures of Nano-Metal-Oxides (nCeO_2_, nMgO and nFe_3_O_4_) and Sulfadiazine

**DOI:** 10.3390/nano9050712

**Published:** 2019-05-07

**Authors:** Fan Zhang, Nan Ye, Se Wang, Yue Meng, Hao Fang, Zhuang Wang, De-Gao Wang

**Affiliations:** 1 School of Environmental Science and Engineering, Collaborative Innovation Center of Atmospheric Environment and Equipment Technology, Jiangsu Key Laboratory of Atmospheric Environment Monitoring and Pollution Control, Nanjing University of Information Science and Technology, Nanjing 210044, China; zhangfan_nuist@163.com (F.Z.); yenan_nuist@163.com (N.Y.); wangse@nuist.edu.cn (S.W.); mengyue-1208@163.com (Y.M.); fh@nuist.edu.cn (H.F.); 2 College of Environmental Science and Engineering, Dalian Maritime University, Dalian 116026, China

**Keywords:** nanoparticles, sulfadiazine, mixture toxicity, freshwater algae, dissolved organic matter

## Abstract

Joint biomarker responses, oxidative stress and membrane systems, were determined for nano-metal-oxides (nMeO, i.e., nCeO_2_, nMgO, and nFe_3_O_4_) and sulfadiazine (SDZ) exposed at relevant low concentrations to two freshwater microalgae *Scenedesmus obliquus* and *Chlorella pyrenoidosa*. The impacts of dissolved organic matter (DOM) on the joint biomarker responses were also investigated. Results indicated that the presence of SDZ significantly decreased the level of intercellular reactive oxygen species (ROS) in the algal cells exposed to each nMeO. Reduction of cell membrane permeability (CMP) and mitochondrial membrane potential (MMP) in the algal cells was observed when the algae were exposed to the mixture of SDZ and the nMeO. The degree of reduction of the ROS level, CMP, and MMP significantly went down with the addition of DOM to a certain extent. Changes in cellular oxidative stress and membrane function depended on the types of both nMeO and algal species. This contribution provides an insight into the hazard assessment of a mixture consisting of emerging contaminants and DOM, as they can coexist in the aquatic environment.

## 1. Introduction

Environmental contaminants in the form of mixtures commonly exist in field-relevant conditions [1,2]. In recent years, the interaction between emerging pollutants, such as engineered nanoparticles (ENPs) [3,4,5] and antibiotics [6,7], have drawn a lot of attention due to their potential ecological toxicity. Upon release or emission, ENPs may interact with antibiotics in the environment, potentially resulting in a co-exposure of organisms and the occurrence of mixture effects [8,9,10]. However, current knowledge about the combined effects of ENPs and antibiotics on ecological species is extremely limited.

Dissolved organic matter (DOM), a ubiquitous abiotic factor, has been demonstrated to play a vital role in the behavior and effect of ENPs in the aquatic environment [11,12,13,14,15,16]. Wang et al. [13] found that DOM alleviated the toxicity of nano-silver to aquatic organisms of different trophic levels. Zhang et al. [17] also observed that DOM reduced the toxicity of materials of the graphene family to freshwater microalgae. However, Ye et al. [18] found that DOM and nano-Al_2_O_3_ synergistically caused cellular responses in freshwater microalgae. Therefore, it is important to take into account the presence of DOM when co-exposure of ENPs and antibiotics is being studied.

nCeO_2_, nMgO, and nFe_3_O_4_ are versatile nano-metal-oxide (nMeO) ENPs that have been widely utilized in many fields [19,20,21]. The importance of evaluating the toxicity of nCeO_2_ [22,23] and nFe_3_O_4_ [24,25] to ecological species has received extensive attention, but aquatic toxicity data for nMgO are comparably rare. Sulfadiazine (SDZ) is a frequently detected antibiotic [26]. Understanding the fate and transport of sulfadiazine in the environment is imperative to its ecological and health risk assessment [27]. The overall objective of this work was to elucidate the impacts of DOM on the aquatic toxicity of the three nMeO and SDZ. Two commonly used freshwater algae species *Scenedesmus obliquus* [28,29] and *Chlorella pyrenoidosa* [30,31] were selected as model organisms. The physicochemical properties of the ENPs in single, binary, and ternary systems were characterized to evaluate their stability in the test medium. Biochemical assays were performed to determine the algal cellular oxidative stress and the membrane function responses, firstly by using one ENP and then mixtures, at relevant low concentrations.

## 2. Materials and Methods

### 2.1. Test Materials, Test Medium, and Test Species

nCeO_2_ (primary size <50 nm), SDZ (purity 98%), and DOM (fulvic acid ≥90%) were purchased from Aladdin Industrial Co. (Shanghai, China). nMgO (primary size ca. 20 nm) and nFe_3_O_4_ (primary size 3–8 nm) were purchased from PlasmaChem GmbH (Berlin, Germany). Ye et al. [18] described the chemical structure of the DOM used in this work in a previous study. A total organic carbon analyzer (Shimadzu Corporation, Kyoto, Japan) was employed to determine the dissolved organic carbon content of the DOM stock solution. Stock suspensions (5 g/L) of the nMeO were freshly prepared in ultra-pure water and sonicated for 30 min in a temperature controlled (25 °C) KH-3200DE water-bath sonicator (Kunshan Hechuang Ultrasonic Instrument Co., Kunshan, China) operated at 150 W with 100% energy input. The stock solution of 0.1% v/v SDZ was prepared in analytical grade dimethylsulfoxide (DMSO). *S. obliquus* and *C*. *pyrenoidosa* were obtained from the Institute of Hydrobiology of the Chinese Academy of Sciences (Wuhan, China) and aliquots were prepared in the algae medium [32]. The pH values of all the treatments were adjusted to 7.8 ± 0.2 prior to testing. For the physicochemical characterizations and toxicity tests, 1 mg/L of nMeO (representing a relevant low concentration [33,34]) or SDZ and 1 mgC/L of DOM was used.

### 2.2. Physicochemical Analysis

The particles in the algae medium were characterized by using a transmission electron microscope (TEM, JOEL 2100f, JOEL Ltd., Tokyo, Japan). The suspensions of nMeO were characterized after being allowed to settle for 0 and 96 h under the same conditions used in the toxicity tests. A ZetaSizer (Nano ZS90, Malvern Instruments Ltd., Worcestershire, UK) was applied to measure the zeta-potential (ZP) and the intensity averaged hydrodynamic diameter (*D*_H_) of the nMeO in the algae medium, based on the phase analysis light scattering techniques and dynamic light scattering, respectively. The concentration of the dissolved metal ions was measured using an inductively coupled plasma optical emission spectrometer (Optima 7000DV Perkin Elmer, Waltham, MA, USA), where the detection limits estimated by calculating the standard deviation of the blank were 20, 20, and 2 µg/L for the elements Ce, Mg, and Fe, respectively. To obtain the ionic phase, the nMeO suspensions were centrifuged at 15,000 rpm for 30 min at 4 °C using a D3024 high-speed micro-centrifuge (Scilogex, Rocky Hill, CT, USA), and then the supernatant was filtered using a 0.02 µm pore diameter syringe filter (Antop 25, Whatman).

### 2.3. Algae Growth, Reactive Oxygen Species, and Membrane Function Assays

Algal growth inhibition tests were conducted as described in our previous study [35]. Briefly, the test solutions were inoculated with 3 × 10^5^ (*S. obliquus*) and 4 × 10^5^ (*C*. *pyrenoidosa*) algal cells mL^−1^. The algae cells were examined under a microscope to check for culture density and normal morphology. The algae used for toxicity tests were maintained at 24 ± 1.0 °C with a photoperiod of 12 h of light (3000–4000 L) and 12 h of dark in climatic chambers, and were shaken manually three times a day. After 96 h (*S. obliquus*) and 72 h (*C*. *pyrenoidosa*), the algal cell density measure was used to calculate the specific growth rate (%).

To measure the intracellular reactive oxygen species (ROS), the 96 h (*S. obliquus*) and 72 h (*C*. *pyrenoidosa*) incubated algal cell suspensions were centrifuged by using the D3024 high-speed micro-centrifuge for 10 min at 15,000 rpm and 4 °C. After discarding the supernatant, a fluorescent probe 2’,7’-Dichlorodihydrofluorescein diacetate (DCFH-DA) purchased from Macklin Biochemical Co., Ltd. (Shanghai, China) at 10 μM was added to incubate with the algal cells for 30 min in the dark at 25 °C. Subsequently, the samples were washed three times with the algae medium. When intracellular ROS generate, the DCFH from the lipase decomposing of the DCFH-DA in the cells transforms into 2’,7’-dichlorofluorescein (DCF), the fluorescence intensity (FI) is then measured and it indicates the extent of the ROS generation.

Fluorescein diacetate (FDA) and the cationic fluorescent dye rhodamine 123 (Aladdin Industrial Co.) were employed as fluorescent probes to measure cell membrane permeability (CMP) and mitochondrial membrane potential (MMP), respectively. Briefly, 96 h (*S. obliquus*) and 72 h (*C*. *pyrenoidosa*) algal cell suspensions were centrifuged at 15,000 rpm for 10 min at 4 °C and were incubated with 10 μM FDA or 10 μM rhodamine 123 in the dark at 25 °C for 30 min, followed by three washes with algae medium. The FI was measured by a fluorescence spectrophotometer (F96PRO, Shanghai Kingdak Scientific Instrument Co., Ltd., Zhejiang, China) with an excitation wavelength of 485 nm and an emission wavelength of 530 nm.

All data are expressed as mean ± standard deviation (SD). Statistically significant differences were determined by the student’s *t*-test (significance level *p* < 0.05).

## 3. Results and Discussion

### 3.1. Physicochemical Characterizations

**Figure 1:**
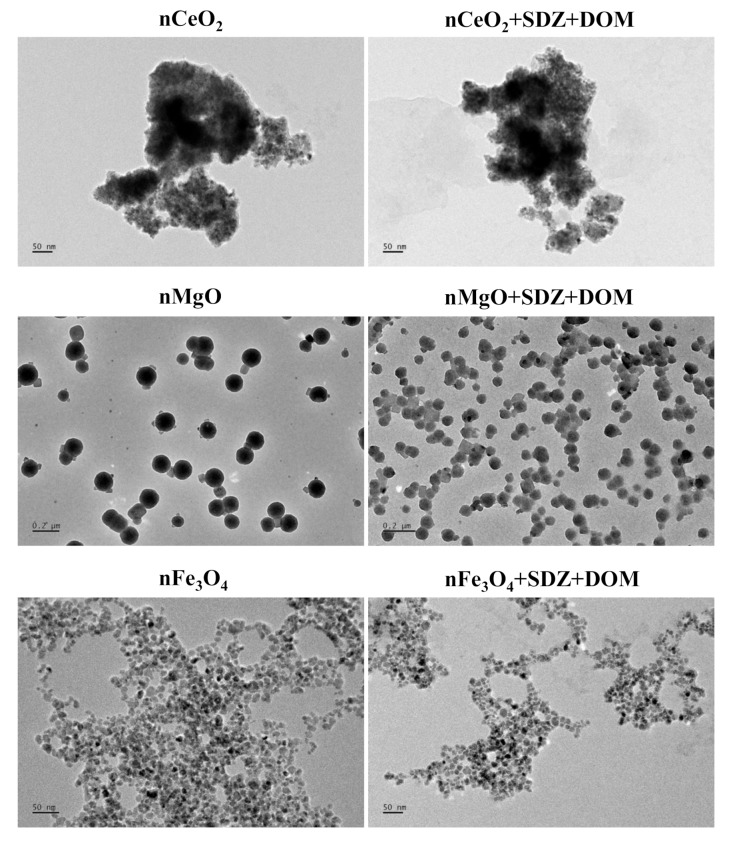
TEM images of the particles in the single and combined systems in the algae medium.

The TEM images show the morphology of the studied particles in the single and combined mixture systems in the algae medium (Figure 1). Analysis of the TEM images indicates that nCeO_2_ agglomerated intensely and formed irregular shapes. Moreover, nCeO_2_ showed greater tendency to agglomerate than the other two particles. nMgO in the single and combined systems were spherical particles with dispersion uniformity. Compared with nMgO in the single systems, the particles in the combined systems showed smaller size and increased density. nFe_3_O_4_ showed spherical structure and also agglomerated in the test medium. Furthermore, the presence of SDZ and DOM obviously alleviated the extent of agglomeration of nFe_3_O_4_. Wang et al. [36] also demonstrated that DOM promoted the suspension of nFe_3_O_4_ by hydrophobic interactions.

**Figure 2:**
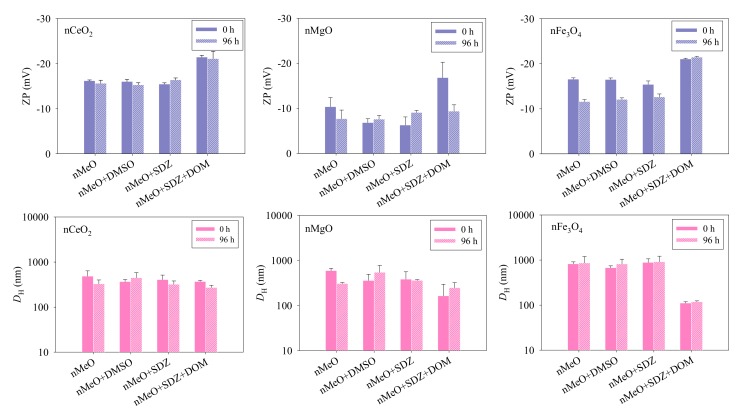
Zeta-potential (ZP) and hydrodynamic diameter (*D*_H_) of the particles in the single and combined systems in the algae medium (pH = 7.8). Data are means ± SD (*n* = 3).

The changes in ZP and *D*_H_ for nCeO_2_, nMgO, and nFe_3_O_4_ with time in the algae medium were measured and the results are shown in Figure 2. The ZP values of the three nMeO were found to be <0, indicating a negative surface charge upon the particles, which could mean that the nMeO particles were stabilized by a surface layer of anions from the test medium. It can be observed that the nMeO showed no obvious changes in the ZP when the particles were suspended in the presence of DMSO and SDZ. However, the ZP of the nMeO particles in the presence of DOM was more negative than when DOM was absent. Moreover, the ZP values of the DOM alone treatments were found to be −10.2 ± 0.8 (0 h) and −11.1 ± 0.8 (96 h), which were generally more positive than the ZP values of the ternary mixtures. This implies that the DOM effects can be explained by the interaction between DOM and the particles rather than the DOM only. The enhanced surface charges might induce adsorption of DOM onto the surface of the particles [11], suggesting that electrostatic repulsion may be the mechanism for dispersion stability. Note that the ZP values of nMgO + SDZ + DOM, nFe_3_O_4_, nFe_3_O_4_ + DMSO, and nFe_3_O_4_ + SDZ systems at 96 h were more positive than their corresponding ZP values at 0 h. This could mean that Mg^2+^- or Fe^3+^-ions released and could have neutralized the negative charges at the surface of the particles.

It was also found that the presence of DMSO and SDZ had no obvious influence on the *D*_H_ values of the nMeO particles (Figure 2). However, the *D*_H_ values of the particles in the binary mixtures of nMeO and SDZ decreased to some extent in the presence of DOM. Furthermore, the *D*_H_ values decreased when DOM was present in the order nCeO_2_ > nMgO > nFe_3_O_4_, regardless of the time of incubation. The results of the *D*_H_ values were in agreement with the TEM analysis. The adsorbed DOM could act as a polyelectrolyte or surfactant and thus provide steric repulsion [37]. Together, these findings suggest that the nMeO particles were relatively stable in the single, binary, and ternary mixtures during the incubation.

In nanotoxicological testing, sonication time affecting the dissolution of nMeO particles should be considered [38,39]. In the present study, we investigated the dissolution amount of nMeO particles at the stock suspension concentrations before (0 min) and after 30 min sonication. As shown in Table 1, there was no significant difference in the dissolved fraction of nMeO between the treatments before and after sonication. This means that the concentration of dissolved metal species was stable during the sonication under this study. Pradhan et al. [38] also observed that increasing the sonication time had no effect on the fraction passing through the 20 nm filter for nCu. In addition, note that the dissolved fraction of nFe_3_O_4_ in the stock suspension before the sonication was 38.41%, implying that the dissolved fraction can influence the aquatic behavior and toxicity of nFe_3_O_4_.

### 3.2. Algal Growth Inhibition Toxicity

**Figure 3:**
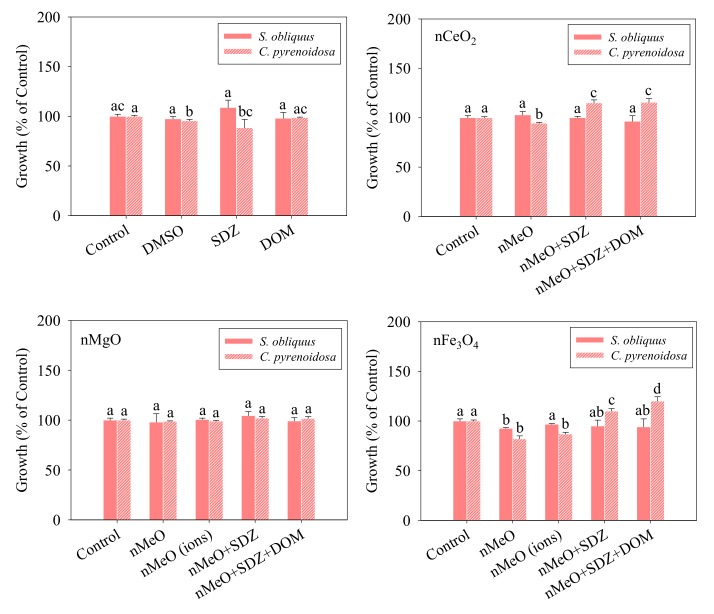
Growth rates of *S. obliquus* and *C. pyrenoidosa* exposed to the treatments. The different letters for each species after exposure to the different treatments indicate the significant differences in growth rates, *p* < 0.05. Data are means ± SD (*n* = 3).

Figure 3 depicts the growth rate of *S. obliquus* after 96 h and *C. pyrenoidosa* after 72 h of incubation for all the treatments at the relevant low concentrations used. The treatments with DMSO and SDZ on their own had no significant inhibition effects on the growth of *S. obliquus*, while the treatments with DMSO and SDZ on their own had significant inhibition effects on the growth of *C. pyrenoidosa*. Moreover, the DOM alone treatment showed no obvious growth inhibition toxicity to the two algae.

For nCeO_2_, the single, binary, and ternary mixtures had no significant inhibition effects on the growth of *S. obliquus*. The growth rate of *C. pyrenoidosa* exposed to the nCeO_2_ alone treatment was lower than the control, while the growth rates of *C. pyrenoidosa* exposed to the nCeO_2_ in the binary and ternary mixtures were higher than the control. The difference of sensitivity to nCeO_2_ might be species-dependent. Moreover, there is no significant difference between the binary and ternary mixtures. That is to say that the presence of SDZ decreased the growth inhibition toxicity of nCeO_2_ to *C. pyrenoidosa*. A previous study indicated the protective role of nCeO_2_ in *Chlamydomonas reinhardtii* and *Phaeodactylum tricornutum* preventing the toxicity of antibiotic erythromycin [40].

For the single, binary, and ternary mixtures of nMgO, no significant inhibition of the algal growth rate was observed. Moreover, the dissolved fractions of nMgO (65 µg/L) showed no growth inhibition toxicity to the algae. Aruoja et al. [41] also reported the nontoxic effect of nMgO on a green alga *Pseudokirchneriella subcapitata* with a median effect concentration >100 mg/L. For nFe_3_O_4_, the treatment with the particles alone inhibited the growth of the two algae, which was consistent with the study by Lei et al. [42] on the algal (*C. pyrenoidosa*) growth inhibition of the particles. Moreover, the dissolved fractions of nFe_3_O_4_ (13 µg/L) showed equivalent growth inhibition toxicity to *C. pyrenoidosa*, implying that the dissolved fractions of nFe_3_O_4_ might contribute to the toxicity to *C. pyrenoidosa*. Similar to nCeO_2_, the binary and ternary mixtures showed no growth inhibition toxicity to the two algae. Note that there is a significant difference between the growth inhibition rates of the binary and ternary mixtures to *C. pyrenoidosa*. This implies that DOM played an important role in modulating the growth of *C. pyrenoidosa* exposed to the binary mixtures of nFe_3_O_4_ and SDZ.

### 3.3. Algal Cellular Oxidative Stress Modulation

**Figure 4:**
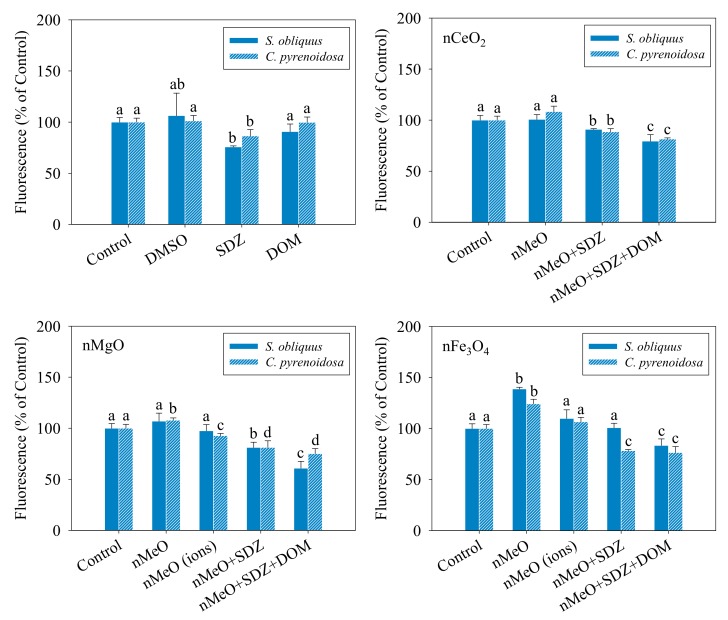
Relative levels of reactive oxygen species in *S. obliquus* and *C. pyrenoidosa* exposed to the treatments. The different letters for each species after exposure to the different treatments indicate the significant differences in fluorescence intensity, *p* < 0.05. Data are means ± SD (*n* = 3).

Figure 4 shows the relative levels of the ROS generated in the *S. obliquus* and *C. pyrenoidosa* cells exposed to the different treatments. Compared to the control, the DMSO and DOM alone showed no effect in the levels of ROS in the two algal cells, while the SDZ alone resulted in an observable decrease. It was observed that nFe_3_O_4_ induced a significant increase in the ROS levels of the two algae, which agrees with the results from Lei et al. [42], where iron-based ENP-induced oxidative stress was the main toxic mechanism to algal toxicity. The consistent trend indicated that the mixtures of SDZ and nMeO decreased the ROS levels, irrespective of the particle types. However, the degree of reduction was comparatively reduced with the addition of DOM. The dissolved fractions of nMgO and nFe_3_O_4_ showed no effects on the levels of ROS, which means that no toxicity or oxidative stress was induced by the dissolved ions. Leung et al. [43] also demonstrated the toxicity of nMgO towards *Escherichia coli* bacterial cells without ROS production for partial nMgO samples. We found that SDZ displayed a protective effect on the algal cells against oxidative stress induced by the particles. Furthermore, the interaction of the nMeO with co-exposed SDZ and DOM reduced the production of ROS. It is undeniable that there are several known limitations of using the fluorescence probe (DCFH-DA), including failure to detect a specific kind of ROS [44,45]. Further studies are needed to expand and confirm the definitive ROS using enzyme-based assays.

### 3.4. Membrane Function Modulation

**Figure 5:**
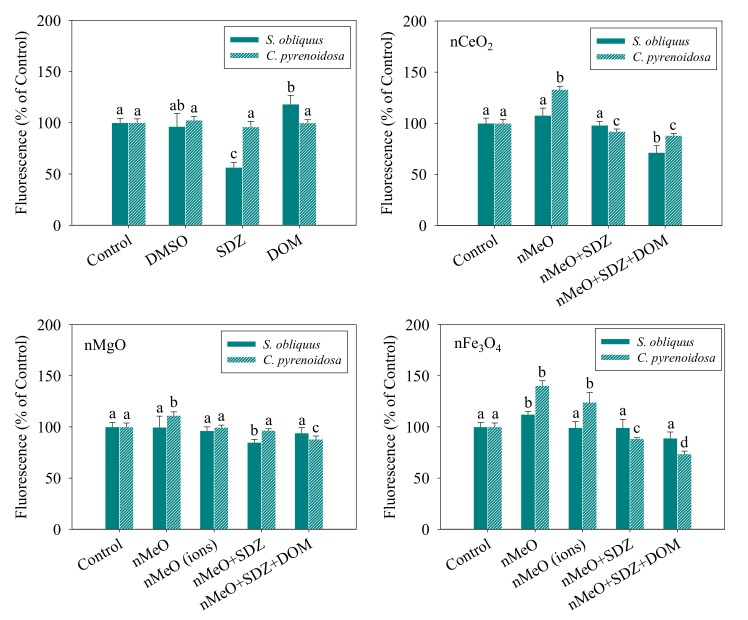
Cellular membrane permeability of *S. obliquus* and *C. pyrenoidosa* exposed to the treatments. The different letters for each species after exposure to the different treatments indicate the significant differences in fluorescence intensity, *p* < 0.05. Data are means ± SD (*n* = 3).

The stability of CMP and MMP is beneficial to maintain the normal physiological function of algal cells [46]. As shown in Figure 5, compared to the control, the DMSO showed no obvious effects on the CMP, while the SDZ alone significantly decreased the CMP of *S. obliquus*, and the DOM alone significantly increased the CMP of *S. obliquus*. The increase in CMP in the algal cells exposed to DOM indicates potential increased risks in the uptake of pollutants, while the decrease observed for the SDZ can be associated with the opposite effect.

For the nCeO_2_, the ternary mixtures remarkably decreased the CMP of *S. obliquus* compared to the individual particles and the binary mixtures, suggesting that the DOM in the ternary mixtures exhibited a distinct effect compared to the DOM alone. The nCeO_2_ alone increased the CMP of *C. pyrenoidosa*, while the binary and ternary mixtures significantly decreased the CMP of *C. pyrenoidosa* in comparison with the control and the alone treatment. Bellio et al. [47] also found that in *Escherichia coli* the outer membrane permeability coefficient increases in presence of nCeO_2_.

For nMgO, the binary mixtures significantly decreased the CMP of *S. obliquus* compared to the control, while the ternary mixtures exhibited no effects on *S. obliquus*. The binary and ternary mixtures containing the nMgO obviously decreased the CMP of *C. pyrenoidosa* compared with the alone treatment. Furthermore, the ternary mixtures contributed more to this decrease in the CMP of *C. pyrenoidosa*. This was similarly observed in the CMP of *C. pyrenoidosa* exposed to the systems containing nFe_3_O_4_. This indicates that DOM plays an important role in modulating the CMP in the algal cells exposed to nMeO. In the case of the nFe_3_O_4_ alone, the CMP increased more than the control. The dissolved fraction of nFe_3_O_4_ affected the CMP of *S. obliquus* less than the nFe_3_O_4_, implying that the nFe_3_O_4_ particles might contribute to the enhancement of the CMP of *S. obliquus*. However, there is no significant difference in the CMP of *C. pyrenoidosa* between the dissolved fraction of nFe_3_O_4_ and the nFe_3_O_4_ alone, suggesting that the dissolved fraction might contribute to the toxicity of the nFe_3_O_4_ to *C. pyrenoidosa* via disturbing the function of CMP.

**Figure 6:**
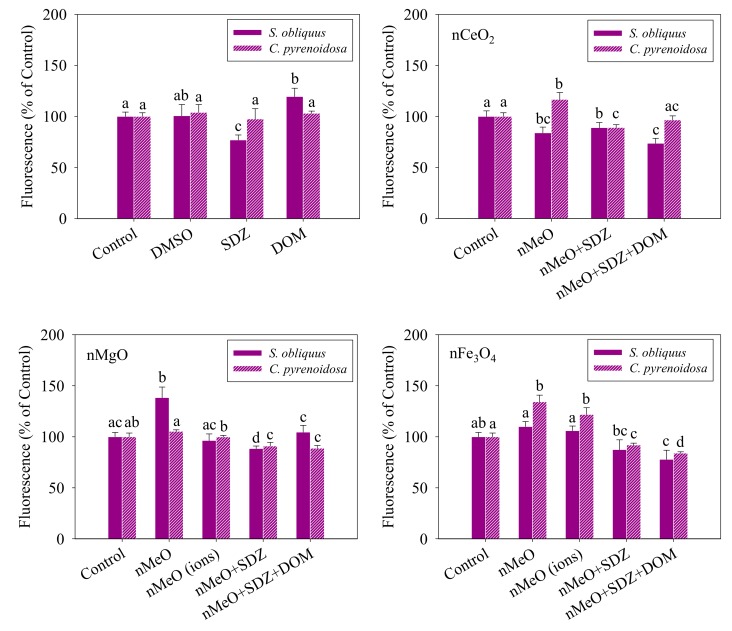
Mitochondrial membrane potential of *S. obliquus* and *C. pyrenoidosa* exposed to the treatments. The different letters for each species after exposure to the different treatments indicate the significant differences in fluorescence intensity, *p* < 0.05. Data are means ± SD (*n* = 3).

As shown in Figure 6, compared to the control, a significant decrease in the MMP of *S. obliquus* cells was observed after 96 h of exposure to the SDZ alone, while the MMP of *S. obliquus* cells was increased by the presence of DOM alone. The changes of the MMP also indicated damage to the mitochondrial function. For the nCeO_2_, the alone treatment posed a completely different effect on the MMP of *S. obliquus* and *C. pyrenoidosa*. Moreover, the presence of DOM decreased the MMP of *S. obliquus* more than the single and binary mixtures of the nCeO_2_. The binary and ternary mixtures significantly decreased the MMP of *C. pyrenoidosa* in comparison with the individual nCeO_2_. Compared to the control, the nMgO only significantly increased the MMP of *S. obliquus*. Leung et al. [43] also demonstrated that the primary mechanism of the death of *E. coli* induced by nMgO was related to cell membrane damage. The binary and ternary mixtures with nMgO significantly decreased the MMP of the two algae compared to the nMgO alone, while the ternary mixtures had less of an effect on the MMP of *S. obliquus* than the binary mixtures. In addition, the dissolved ions shed from the nMgO showed no effect on the MMP. The degree of reduction of the MMP in the algal cells exposed to the nFe_3_O_4_ significantly went down with the addition of SDZ and DOM.

Similar to the results of CMP, there is no significant difference in the MMP of *C. pyrenoidosa* between the dissolved fraction of nFe_3_O_4_ and the nFe_3_O_4_ alone, suggesting that the dissolved fraction might contribute to the toxicity of the nFe_3_O_4_ to *C. pyrenoidosa* via disturbing the mitochondrial function. Compared with the single and binary mixtures, the MMP of *C. pyrenoidosa* decreased more when the DOM was present. This decrease in the MMP might be caused by the depolarization of the mitochondria in the algal cells exposed to the treatments. Together, it is reasonable to believe that changes in the membrane function could be a sensitive cellular indicator to indicate the toxicological effect of xenobiotics.

In the present study, DOM displayed different degrees of impact on the behavior of the three nMeO (nCeO_2_, nMgO, and nFe_3_O_4_) in the test medium, and modulated the cellular oxidative stress and membrane system responses of the two freshwater algae (*S. obliquus* and *C. pyrenoidosa*) to the nMeO when SDZ co-existed. The role of DOM was mainly associated with the types of nMeO and the species of algae. Furthermore, the mechanism of the effects of DOM on the algal cellular responses to the binary mixtures may be associated with their binding with mixture components [48,49]. It is likely that the complexation of DOM to SDZ or the dissolved fraction of the nMeO made them less bioavailable, and the adsorption of DOM upon the nMeO limited the interactions of particles with the algal cells.

## 4. Conclusions

In summary, the presence of SDZ significantly decreased the level of intercellular ROS in *S. obliquus* and *C. pyrenoidosa* induced by the three nMeO. SDZ decreased CMP and MMP levels of *S. obliquus* exposed to the nMeO to some extent. Furthermore, the degree of reduction of ROS, CMP, and MMP significantly went down with the addition of DOM. The results we presented here confirmed that SDZ and DOM helped protect against oxidative stress responses in the algal cells exposed to nMeO, and interrupted the algal cell membrane functions. Our findings demonstrated that DOM played an important role and should not be omitted when evaluating the combined risk of ENPs and antibiotics.

## Figures and Tables

**Table 1:** Dissolved fraction of the nano-metal-oxide (nMeO) particles in the stock suspension concentrations before (0 min) and after 30 min sonication ^a^.

nMeO	Fraction (%)	
	0 min	30 min
nCeO_2_	0.02 ± 0.00	0.03 ± 0.01
nMgO	0.69 ± 0.04	0.81 ± 0.16
nFe_3_O_4_	38.41 ± 3.02	34.34 ± 2.75

^a^ Data are means ± SD (*n * = 2).
